# Reduced growth and biofilm formation at high temperatures contribute to *Cryptococcus deneoformans* dermatotropism

**DOI:** 10.1242/dmm.052141

**Published:** 2025-03-25

**Authors:** Claudia L. Charles-Niño, Gunjan M. Desai, Nicholas Koroneos, Mohamed F. Hamed, Neena Jain, William Lopes, Anthony Braswell, Alexander Linares, Melissa E. Munzen, Joshua D. Nosanchuk, Marilene H. Vainstein, Luis R. Martinez

**Affiliations:** ^1^ Department of Oral Biology, University of Florida College of Dentistry, Gainesville, FL 32610, USA; ^2^ Department of Biomedical Sciences, Long Island University-Post, Brookville, NY 11548, USA; ^3^ Department of Medicine, Reinaissance School of Medicine, Stony Brook University, Stony Brook, NY 11794, USA; ^4^ Department of Pathology (Faculty of Veterinary Medicine), Mansoura University, Mansoura 35516, Egypt; ^5^ Department of Medicine (Division of Infectious Diseases), Albert Einstein College of Medicine, Bronx, NY 10467, USA; ^6^ Centro de Biotecnologia, Universidade Federal do Rio Grande do Sul, Porto Alegre, Rio Grande do Sul 91501-970, Brazil; ^7^ Department of Biology, University of North Florida, Jacksonville, FL 32224, USA; ^8^ Emerging Pathogens Institute, University of Florida, Gainesville, FL 32610, USA; ^9^ Center for Immunology and Transplantation, University of Florida, Gainesville, FL 32610, USA; ^10^ McKnight Brain Institute, University of Florida, Gainesville, FL 32610, USA; ^11^ Center for Translational Research in Neurodegenerative Disease, University of Florida, Gainesville, FL 32610, USA

**Keywords:** *C. deneoformans*, *C. neoformans*, GXM, Heat-shock proteins, Skin infections, Temperature, Thermal tolerance

## Abstract

*Cryptococcus deneoformans* (*Cd*) and *C. neoformans* (*Cn*) differ in geographic prevalence and dermatotropism, with *Cd* strains more commonly isolated from temperate regions and skin infections. Rising global temperatures prompt concerns regarding selection for environmental fungal species with increased thermotolerance, as high mammalian temperatures provide protection against many fungal species. *Cd* and *Cn* strains exhibit variations in thermal susceptibility, with *Cd* strains being more susceptible to higher temperatures. Here, we identified differences in capsular polysaccharide release, adhesion and biofilm formation between strains both *in vivo* and *in vitro*. Histological results suggested that the dermatotropic predilection associated with *Cd* relates to biofilm formation, possibly facilitating latency and extending fungal survival through protection from high temperatures. We demonstrated that *Cn* strains were more tolerant to mammalian and febrile temperatures than *Cd* strains. Similarly, *Cd* strains showed reduced expression of heat-shock protein 60 and 70, after prolonged exposure to high temperature. Our findings suggest that fungal adhesion, biofilm formation, inflammation and thermotolerance contribute to tissue tropism and disease manifestation by *Cn* and *Cd*, supporting the recently assigned species distinction to each of these serotypes.

## Teaser

**Summary:** Differences in the thermotolerance of *Cryptococcus deneoformans* and *Cryptococcus neoformans* influence the host route of infection, explaining dermatotropism in *Cryptococcus deneoformans*.

## INTRODUCTION

*Cryptococcus neoformans* (*Cn*) is an opportunistic fungus that can cause life-threatening infection in individuals with significantly impaired immunity, accounting for ∼112,000 deaths/year globally (Rajasingham et al., 2022). *Cn* serotypes are classified based on antigenic differences resulting from structural variation of the major capsular polysaccharide glucuronoxylomannan (GXM) (Belay et al., 1996; Bhattacharjee et al., 1984; Wilson et al., 1968). Detailed studies on genetic differences led to the distinction of *Cn* into two varieties: *Cn* var. *grubii* (serotype A) and *Cn* var. *neoformans* (serotype D) (Franzot et al., 1999).

There are notable differences in the geographic distribution of *Cn* and *Cryptococcus deneoformans* (*Cd*) isolates obtained from clinical and environmental sources, which might reflect climatic tolerances and differing virulence factors (Bennett et al., 1977; Kwon-Chung and Bennett, 1984). *Cn* predominates in most areas of the world, with the exception of certain northern European countries (Steenbergen and Casadevall, 2000). *Cryptococcus* species reveal wide variation in thermal susceptibility, with *Cd* strains having greater susceptibility to high-temperature killing than *Cn* strains (Martinez et al., 2001).

Human-induced climate change is predicted to raise global temperatures, potentially selecting for thermotolerance among environmental fungi (Casadevall et al., 2019). Interestingly, *Cd* is associated with infection of the skin, potentially due to the lower temperatures relative to core temperatures (Kantarcioglu and Yücel, 2002; Murad and Murphy, 2014; Neuville et al., 2003; Tortorano et al., 1997). The predilection of *Cd* for skin and *Cn* for internal infections raises concerns regarding thermotolerance, as warmer host temperatures confer protection against systemic cryptococcosis (Cordero et al., 2023; Perfect et al., 1980). As the number of extremely hot days increases, it is possible for environmental *Cd* strains, and other non-pathogenic fungi, to adapt to higher ambient temperatures and overcome the host endothermic defense.

Biofilm formation is an important survival strategy in hostile environmental conditions and predation (Joubert et al., 2006; Martinez and Casadevall, 2007). *Cn* and *Cd* can colonize tissues and form biofilm-like structures comprised of yeast cells surrounded by capsular polysaccharide, preventing efficient eradication of the organism (Martinez and Casadevall, 2005). Previous studies revealed *Cd* strain B3501 to produce more robust biofilms than *Cn* strain H99 due to differences in polysaccharide capsule composition and release (Martinez and Casadevall, 2005).

Transcriptomic studies comparing *Cn* H99 and *Cd* B3501 strains after exposure to mammalian body temperature (37°C) revealed increased mRNA expression for several genes encoding heat-shock proteins (HSPs) (Steen et al., 2002). *Cn* HSP70 confers virulence through interactions with human alveolar epithelial cells and by reducing murine macrophage-mediated fungal killing (Silveira et al., 2013). The HSP70 homolog, SSA1, serves as a co-activator of laccase expression, the enzyme responsible for melanization, in *Cd* strain JEC21 (Zhang et al., 2006). The role of HSP60 is not well known; however, functional differences between *Cn* and *Cd* strains could be critical for survival during environmental stress and infection.

Given their association with cutaneous involvement (Neuville et al., 2003) and reduced thermotolerance (Martinez et al., 2001), we hypothesized that *Cd* strains have predilection for skin colonization. To test this hypothesis, we compared the ability of *Cn* H99 and *Cd* B3501 strains to survive, form biofilm-like aggregates and persist during inflammation in cutaneous tissue using a mouse model of excisional wound. Additionally, we compared the thermotolerance and differential expression of *hsp60* and *hsp70* in multiple *Cn* and *Cd* clinical strains to explain the dermatotropism observed in *Cd* strains and the ability of *Cn* strains to cause systemic disease. Our study provides data on the differential pathogenesis of *Cn* and *Cd*, as well as insight into thermodynamic responses, which might contribute to the development of better strategies for managing infections.

## RESULTS

### *Cd* delays wound healing and forms biofilm-like structures *in vivo*

**Fig. 1.**
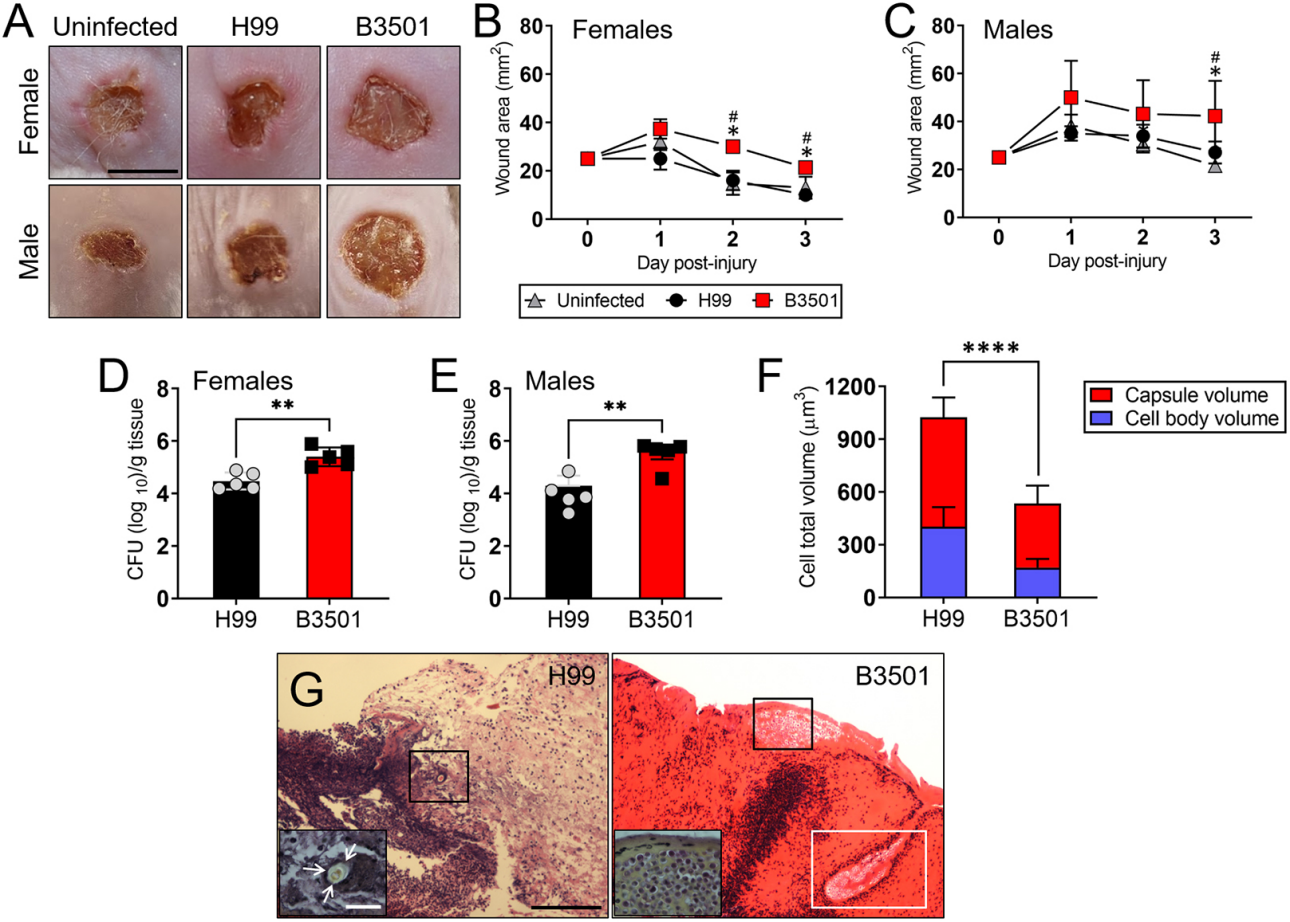
***C. deneoformans* (*Cd*) delays wound healing in female and male mice.** (A) Wounds of uninfected or *C. neoformans* (*Cn*) strain H99- or *Cd* strain B3501-infected female and male Balb/c mice, 2 days post-wounding. Scale bar: 5 mm. (B,C) Wound size analysis of female (B) and male (C) Balb/c mouse skin lesions. Time points are the averages of the results for five different wound measurements (*n=*5 mice per group; a single wound per mouse), and error bars denote s.d. Significance (*P*<0.05) was calculated by multiple Student's *t*-test analysis. ‘*’ and ‘^#^’ indicate significantly higher than uninfected and H99-infected groups, respectively. (D,E) Wound fungal burden (CFU, colony-forming units) in female (D) and male (E) Balb/c mice with wounds infected with 10^7^ *Cn* H99 or *Cd* B3501 (*n=*5 per group). At 3 days post-infection (dpi), infected skin tissue was removed from mice, and fungal burden was determined. Bars are the averages of the results for five different wound CFU measurements, and error bars denote s.d. Each symbol (gray circles or black squares) represents CFU for individual wound tissue homogenates. (F) Cell body and capsule volume measurements for each cryptococcal strain were determined in wounded tissue excised from female mice by India ink suspensions and light microscopy. The volume of the capsule was calculated using the equation for the volume of a sphere, 4/3 π (*D*/2)^3^, such that the capsule volume (*V*_c_) was the difference between the whole cell volume (*V*_wc_) and the volume of the cell body (*V*_cb_). Bars represent cell total volume (red, capsule volume; blue, cell body volume) average for 50 cryptococci. Error bars denote s.d. For D-F, significance (****P<0.0001; **P<0.01) was calculated by paired two-tailed Student's *t*-test. (G) Histological analysis of female Balb/c mice infected with *Cn* H99 (left) or *Cd* B3501 (right), day 3. Representative Hematoxylin and Eosin (H&E)-stained sections of the skin lesions are shown (20× magnification), with the insets (40×, higher magnification of the black line boxes) showing mucicarmine staining for fungal cells (white arrows). The white line box in B3501-infected tissue indicates a hair follicle filled with cryptococci. Scale bars: 100 μm and 10 μm (insets).

*Cn* and *Cd* differed in dermatotropism, with higher colony-forming units (CFUs) of *Cd* strains being recovered from infected skin (Dromer et al., 1996). We used a mouse skin model of infection (Balb/c females and males; *n*=5 animals per sex per group) to compare *Cd* B3501 and *Cn* H99 pathogenesis (Fig. 1). We specifically monitored wound healing for 3 days post-injury or infection (dpi) to assess early host responses to the cryptococcal species. Mice of both biological sexes infected with *Cd* B3501 [female (2 dpi) and male (3 dpi)] exhibited larger eschar (dry, dark scab) sizes after wounding compared with *Cn* H99-infected mice (Fig. 1A). In female mice at 2 dpi (Fig. 1B), the eschar area of B3501-infected wounds reached ∼30 mm^2^, whereas the eschars from the uninfected (*P<*0.05) and H99 (*P<*0.05) groups were each ∼15 mm^2^. At 3 dpi (Fig. 1B), the eschars from B3501-infected wounds measured ∼21 mm^2^, whereas the eschar sizes in the uninfected (*P<*0.05) and H99 (*P<*0.05) groups had decreased to ∼13 and 9 mm^2^, respectively. In male mice (Fig. 1C), although there was increased wound size in B3501-infected animals compared to that in the uninfected and H99-infected groups at 2 dpi, these differences were not statistically significant. In contrast, the eschar area of B3501-infected wounds reached ∼42 mm^2^, whereas the eschar areas in the uninfected (*P*<0.05) and H99-infected (*P*<0.05) groups decreased to ∼22 and 27 mm^2^, respectively. Fungal dissemination to other sites was not observed in this murine model following infection with these cryptococcal strains. These *in vivo* studies demonstrate that infection with *Cd* B3501 resulted in slower wound closure rates compared to infection with the *Cn* H99 strain.

Moreover, we compared wound healing between female and male mice under the different experimental conditions: uninfected (Fig. S1A) or infected with H99 (Fig. S1B) or B3501 (Fig. S1C). Uninfected male animals demonstrated significantly slower wound healing compared to their female counterparts at ≥2 dpi (*P*<0.05). Similarly, male mice infected with either H99 (*P<*0.05; Fig. S1B) or B3501 (*P<*0.05; Fig. S1C) exhibited significantly reduced wound healing compared to female mice infected with the same strains at 3 dpi. These findings suggest that sex differences in wound healing in Balb/c mice are exacerbated by cryptococcal infection.

The prevalence of *Cn* and *Cd* in infected mice was further explored by investigating the fungal burden in the infected skin tissue. Female and male mice were euthanized at 3 dpi. Homogenates from wounded skin tissue revealed that, in both sexes, B3501-infected mice exhibited a significantly higher microbial burden than that of the H99-infected mice (*P*<0.01; Fig. 1D,E). The fungal cell body volume and capsule of yeast cells recovered from infected wounds from female mice were measured to assess potential changes in cellular and capsular morphology during infection (Fig. 1F). The cell body and capsule size of the *Cn* H99 strain were significantly larger than those of the B3501 (*P>*0.0001) strain (Fig. 1F).

Histological examinations using Hematoxylin and Eosin (H&E) and mucicarmine (MC) stains revealed that tissue from wounds of H99-infected mice exhibited intense cellular infiltrates, with occasional individual large yeast cells (Fig. 1G, left inset, white arrows) that were similarly distributed throughout the epidermal and dermal layers (Fig. 1G, left panel). Analysis of B3501-infected tissue revealed a more localized, intense cellular infiltration, with large numbers of fungal cells in a biofilm-like structure (Fig. 1G, right inset). These biofilm-like cells were associated with the stratum corneum layer of the epidermis (Fig. 1G, right panel, black rectangle) and hair follicles (Fig. 1G, right panel, white rectangle).

These data suggest that *Cd* strain B3501 forms a biofilm-like structure near the epidermis or along hair follicles, likely due to the cooler temperature at the cutaneous tissue surface and reduced inflammatory response.

### *Cd* induces less robust neutrophil infiltration compared to *Cn*

**Fig. 2.**
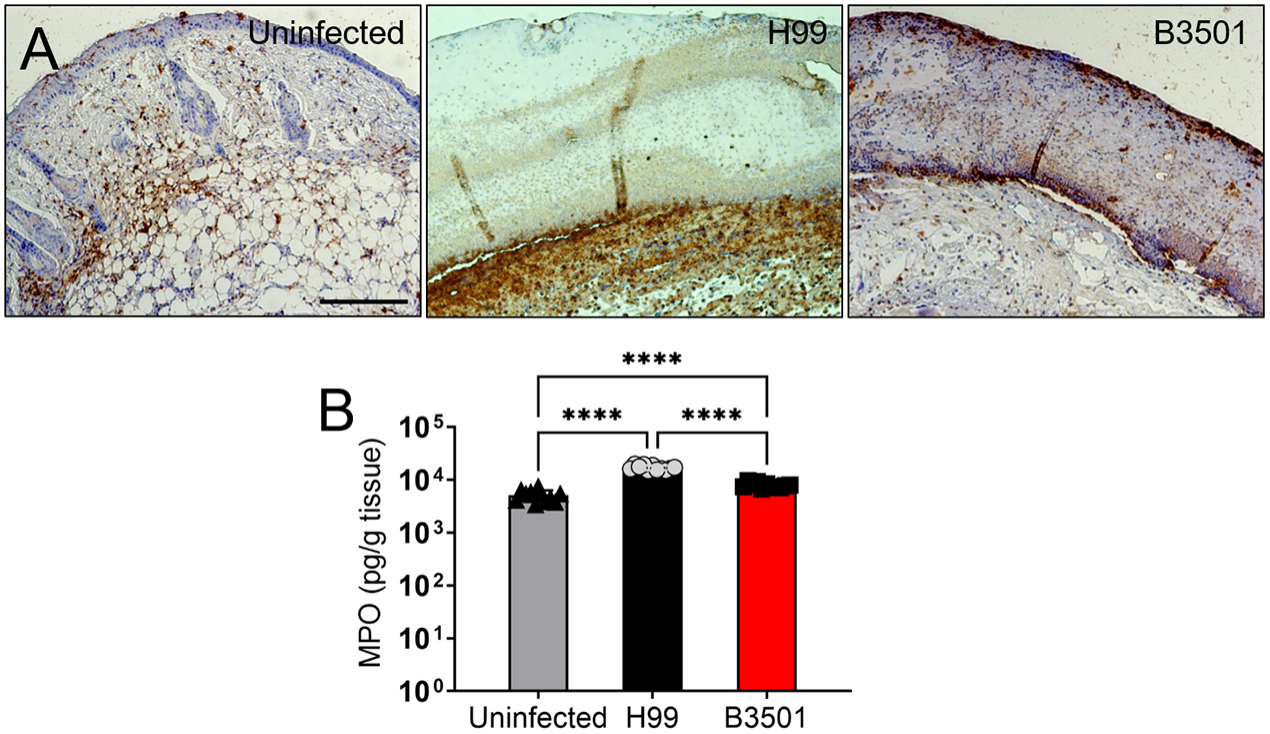
***Cd* stimulates milder neutrophil response to the wounded tissue than *Cn*.** (A) Immunohistochemistry of myeloperoxidase (MPO) released by neutrophils in wounds infected with *Cn* H99 (middle) or *Cd* B3501 (right) strains or uninfected tissue (left). MPO-specific monoclonal antibody (mAb) was used to stain MPO (brown) released in skin tissue. Representative MPO-immunostained sections (20× magnification) of the skin lesions are shown. Scale bar: 100 µm. (B) MPO concentration in the supernatant of *Cn*- and *Cd*-infected tissue homogenates of mice (*n=*5 per group). Each symbol (black triangle, gray circle or black square) represents a measurement for an individual mouse. Bars represent the mean values; error bars denote s.d. Significance (****P<0.0001) was calculated by one-ANOVA and adjusted by Tukey's post-hoc analysis.

Neutrophils play an important function in modulating the development of the immune response. Myeloperoxidase (MPO) is released by neutrophils and is indicative of the number of these cells at a site of infection (Khan et al., 2018; Kothari et al., 2011). Therefore, we used MPO staining as a quantitative assay to determine neutrophil infiltration in *Cn*- compared to *Cd*-infected skin (Fig. 2). Tissue from wounds of H99-infected mice had increased neutrophil-rich infiltrates mostly associated with the dermal and subcutaneous layers compared with that from B3501-infected mice (Fig. 2A). Similarly, homogenates showed significantly higher levels of MPO in skin from H99-infected mice compared to that from uninfected mice (*P<*0.0001) or B3501-infected mice (*P<*0.0001) (Fig. 2B). Notably, although *Cd* B3501 strain induced significantly higher MPO concentrations in infected mice than in uninfected mice (*P<*0.05), the levels of the enzyme were substantially lower than those observed in H99-infected mice. These results not only validate the findings from the MPO immunostaining (Fig. 2A) but also suggest that the persistence of *Cd* B3501 strain in cutaneous tissue is associated with a significantly lower inflammatory response.

### *Cd* and *Cn* strains differ in the distribution of GXM released in wounds, highlighting distinct infection patterns in mouse skin tissue

**Fig. 3.**
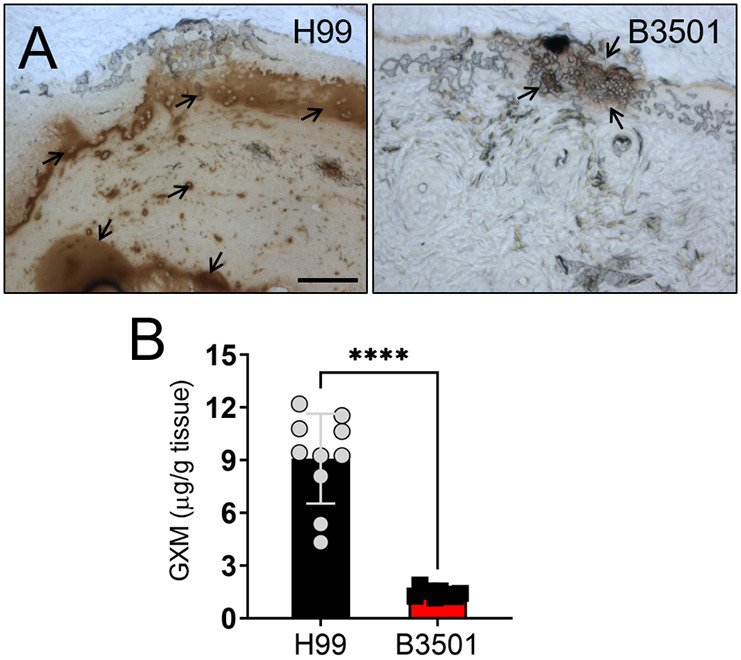
***Cn* and *Cd* strains differ in the distribution of glucuronoxylomannan (GXM) released in mouse wounds.** (A) Histological analysis of GXM released by *Cn* H99 (left) and *Cd* B3501 (right) strains in wounds of infected Balb/c mice. GXM-specific mAb 18B7 (mAb 18B7) was used to stain capsular polysaccharide (brown; black arrows) released in skin tissue. Representative GXM-immunostained sections of the skin lesions (20× magnification) are shown. Scale bar: 100 µm. (B) GXM concentration in the supernatant of *Cn*- and *Cd*-infected tissue homogenates of mice (*n=*10 per group). Each symbol (gray circle or black square) represents an individual GXM determination per mouse. Bars represent the mean values; error bars denote s.d. Significance (****P<0.0001) was calculated by paired two-tailed Student's *t*-test.

The distribution of capsular polysaccharide released by *Cryptococcus* strains in skin tissue was investigated by visualizing the staining with a specific monoclonal antibody (mAb) against GXM (Casadevall et al., 1998) (Fig. 3). In *Cn* H99-infected tissue, copious amounts of GXM were released (Fig. 3A, left panel, black arrows) and distributed throughout the infected tissue (Fig. 3A, left panel). Individual fungal cells were observed scattered diffusely throughout the epidermal and dermal tissue. However, the *Cd* B3501 strain exhibited localized GXM release in the epidermis (Fig. 3A, right panel). Additionally, the biofilm-like structure in B3501-infected tissue revealed adjacent darker localized regions of mAb-stained GXM, in which yeast cells were densely packed within a surrounding layer of polysaccharide (Fig. 3A, right panel, black arrows). GXM was extensively released by *Cn* during infection. To quantify the amount of GXM released by H99 and B3501 strains, we performed a capture enzyme-linked immunosorbent assay (ELISA) on tissue homogenates (Fig. 3B). The H99 strain released significantly higher amounts of GXM than the B3501 (*P<*0.0001) strain. Our results demonstrated differential GXM secretion in murine cutaneous tissue between the *Cn* H99 and *Cd* B3501 strains.

### *Cd* and *Cn* stimulate different cytokine expression profiles by the host

We measured the cytokine response in the wounds of mice intradermally infected with *Cd* B3501 or *Cn* H99, focusing primarily on cytokines involved in inflammation and the host acute response. At 3 dpi, wound tissues from mice infected with H99 exhibited significantly higher concentrations of pro-inflammatory TNF-α (also known as TNF), IFN-γ, IL-1β and IL-6 than tissues from mice infected with B3501 or uninfected naïve mice (*P<*0.05; Table S1). Along with the MPO results, these data suggest that intradermal infection by the *Cn* H99 strain induces a stronger inflammatory immune response than that by the *Cd* B3501 strain.

### *Cd* significantly adheres to human skin cells

We observed that the B3501 strain forms biofilm-like structures *in vivo* and that surface adhesion is a crucial stage in microbial biofilm formation. To further investigate, we examined the ability of B3501 and H99 strains to adhere to model human skin using keratinocyte-like CCD-1106 KERTr and melanoma SK-MEL-28 cells (Fig. S2). Using fluorescent microscopy (Fig. S2A) and cell counts (Fig. S2B), we demonstrated that *Cd* B3501 cells adhered to CCD-1106 KERTr cells in higher numbers than *Cn* H99 cells (*P*<0.0001) after 2 h incubation. *cap67* is a mutant strain defective in capsular production derived from the *Cd* B3501 strain (Xiao et al., 2008) and was used as a negative control to demonstrate the importance of the capsule in adhesion. CFU determinations showed that *Cd* B3501 cells displayed stronger adhesion properties to SK-MEL-28 melanoma cells than the *Cn* H99 (*P*<0.0001) or *cap67* (*P*<0.0001) cells after 2 h incubation (Fig. S2C). Furthermore, we investigated whether capsular polysaccharide variations between *Cn* and *Cd* cells impacted melanoma cell adhesion and compared clinical isolates of each species (Fig. S2D). There were no differences in skin cell adhesion between the species. These results validate that functional capsular production is important in the successful adhesion of fungal cells to a biological surface (Martinez and Casadevall, 2005). In contrast, although *Cn* H99 and *Cd* B3501 cells differed in adhesion to skin cells, this observation is strain specific and does not necessarily translate to a species level considering the adhesion variations exhibited by each species’ clinical isolates.

### *Cd* cells displayed lower thermotolerance than *Cn* cells

**Fig. 4.**
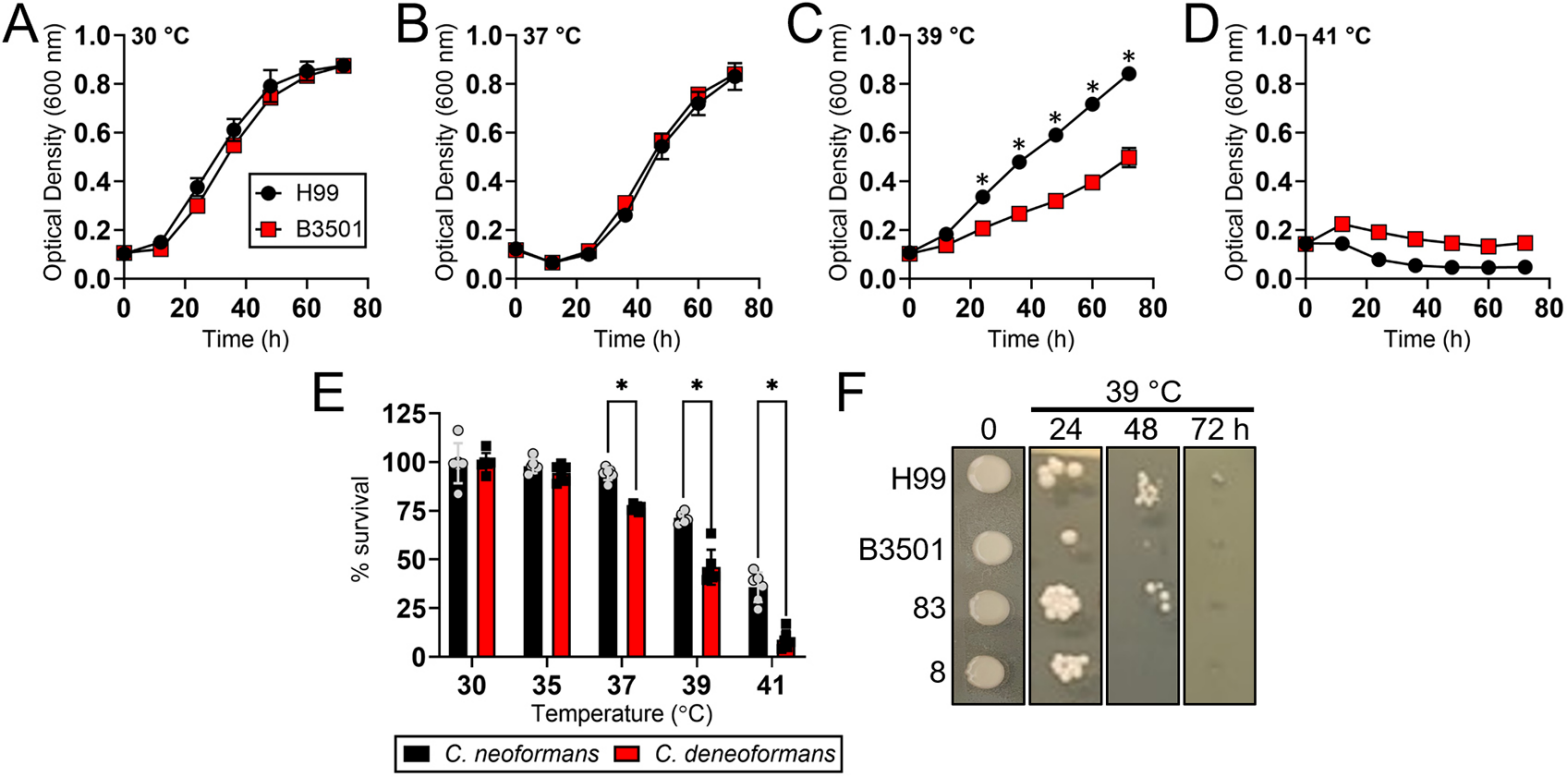
***Cd* strains are more susceptible to high temperature than *Cn* strains.** (A-D) Growth differences between *Cn* H99 and *Cd* B3501 strains at 30°C (A), 37°C (B), 39°C (C) and 41°C (D) were determined using Bioscreen C analysis. Each time point represents the average of the results for four measurements, and error bars denote s.d. (E) Percentage survival of *Cn* and *Cd* strains after exposure to increasing temperatures (30, 35, 37, 39 and 41°C) for 30 min. Bars represent the average of six different strains per species (gray circles and black squares), and error bars denote s.d. Significance (*P<0.05) was calculated using multiple Student's *t*-test analysis. (F) Growth of *Cn* (H99 and 83) and *Cd* (B3501 and 8) strains after exposure to 39°C for 72 h. These experiments were performed twice, and similar results were obtained.

We compared the thermotolerance of the *Cryptococcus* strains H99 and B3501 to elevated temperatures over 72 h using a Bioscreen C system (Fig. 4). Because *Cryptococcus* species grow optimally at 30°C, we used this temperature as the control. Both cryptococcal strains exhibited similar growth curves at 30°C (Fig. 4A) and 37°C (Fig. 4B). However, at mammalian body temperature (37°C), their growth showed a delayed logarithmic phase compared to growth at optimal (30°C) temperature. *Cn* H99 cells displayed a higher growth rate than *Cd* B3501 cells at 39°C, indicating greater thermotolerance (Fig. 4C). Neither strain grew well at 41°C (Fig. 4D), a temperature selected for our studies to test the highest range for fever in mammals.

We have previously demonstrated that *Cn* strains are more thermotolerant than *Cd* strains (Martinez et al., 2001). However, we did not explore the possible mechanistic explanations of these observed differences in thermotolerance. As proof of concept, we expanded our studies beyond the *Cn* H99 and *Cd* B3501 strains, by testing several additional strains of each species (*n*=7; Fig. 4E). First, we performed multilocus sequence typing (MLST) to confirm that our sample represents a reasonable range of genetic diversity within each clade (Table S2). Next, we exposed all the strains to various temperatures (30, 35, 37, 39 and 41°C) for 30 min and determined the differential survival between *Cn* and *Cd* strains (Fig. 4E). We did not observe any survival differences between strains from both cryptococcal species after exposure to 30°C and 35°C temperature. In contrast, *Cn* strains, on average, demonstrated significantly higher thermotolerance than *Cd* strains following exposure to mammalian body temperature (37°C, *P*<0.0001) or higher temperatures (39°C, *P*<0.001; 41°C, *P*<0.0001) (Fig. 4E). Furthermore, we evaluated and compared the growth of *Cn* (H99 and 83, clinical isolate) and *Cd* (B3501 and 8, clinical isolate) strains at 39°C for 72 h on Sabouraud dextrose agar plates (Fig. 4F). *Cn* strains demonstrated significantly higher thermotolerance than *Cd* strains 24 h post-exposure to 39°C. These findings confirm that *Cn* strains adapt better to higher temperatures than *Cd* strains, which might explain the *Cd* tropism for cooler tissues, such as the sinuses or skin.

### *Cn* cells induce greater increase in capsule size at higher temperature than *Cd* cells

**Fig. 5.**
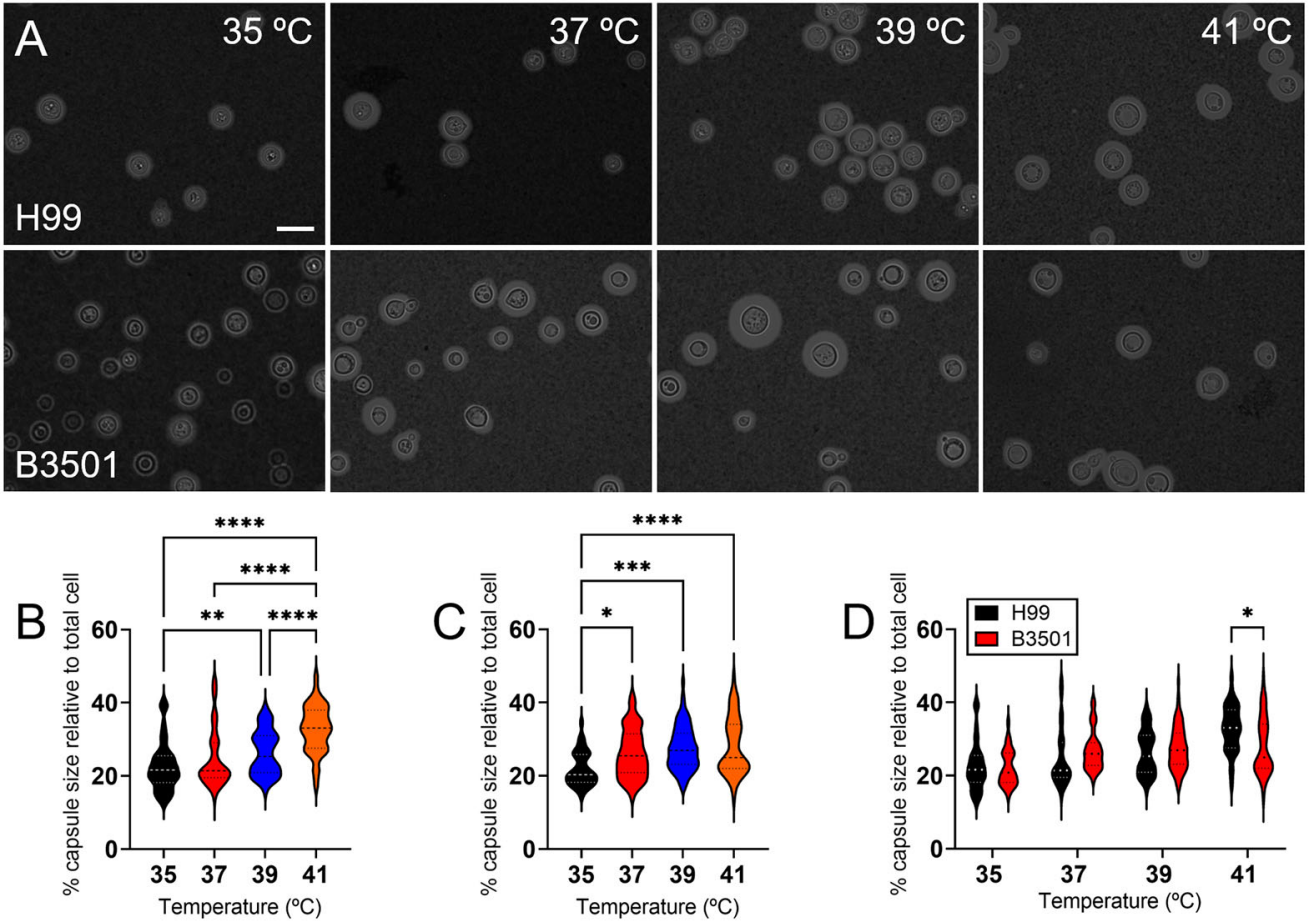
***Cn* and *Cd* show different capsule growth induction in response to increasing temperature.** (A) India ink staining images of *Cn* H99 (top row) and *Cd* B3501 (bottom row) strains after exposure to increasing temperatures (35, 37, 39 and 41°C) for 6 h. The pictures were taken using a 40× power field. Scale bar: 20 µm. (B,C) The percentage capsule size relative to total cell measurements of H99 (B) and B3501 (C) strains was measured and compared between the different temperatures. For B and C, violin plots show the averages (*n*≥50 cells per group; dashed lines) and s.d. of the results. Significance (****P<0.0001; ***P<0.001; **P<0.01; *P<0.05) was calculated by one-way ANOVA and adjusted using Tukey's post hoc analysis. (D) Percentage capsule size relative to total cell measurements of *Cn* H99 (B) and *Cd* B3501 (C) were compared. Significance (*P<0.05) was calculated by multiple Student's *t*-test analysis.

To investigate the differences in adaptation of *Cn* H99 and *Cd* B3501 cells to high temperature, we determined the percentage capsule size relative to the total cell size (in volume) after exposure to increasing temperatures (35, 37, 39 and 41°C) for 6 h (Fig. 5). *Cn* cells demonstrated a significant increase in capsule size percentage after exposure to ≥39°C (compared to 35°C, *P*<0.01; Fig. 5A,B). In contrast, *Cd* displayed capsular enlargement at mammalian body temperature (relative to 35°C, *P*<0.05; Fig. 5A-C). There were no differences in capsule size between *Cn* H99 and *Cd* B3501 cells after growth at 35-39°C (Fig. 5D). However, the capsule size of *Cn* H99 cells was significantly larger than that observed in *Cd* B3501 cells at 41°C (*P*<0.05; Fig. 5D). We exposed *Cn* and *Cd* strains to 41°C for 6 h, and measured their viability (Fig. S3), observing no difference between *Cn* and *Cd*. Thus, the differences found in capsule size between *Cn* and *Cd* were dependent on temperature. These results suggest that *Cd* increases its polysaccharide capsule size after exposure to 37°C, which might explain its preference for the colonization of cooler tissues. Although *Cd* strains can invade hotter tissue and therefore cause systemic disease, it is well documented that *Cn* strains are significantly more frequently isolated from systemic infections, especially those involving the central nervous system (CNS), than other species of *Cryptococcus* (Chan et al., 2014; Ibe et al., 2023; Neuville et al., 2003). In this regard, we demonstrated that *Cn* enlarges its capsule size at ≥39°C, which supports this species’ ability to cause systemic infection.

### *Cd* exhibits greater loss of biofilm mass than *Cn* at 39°C

**Fig. 6.**
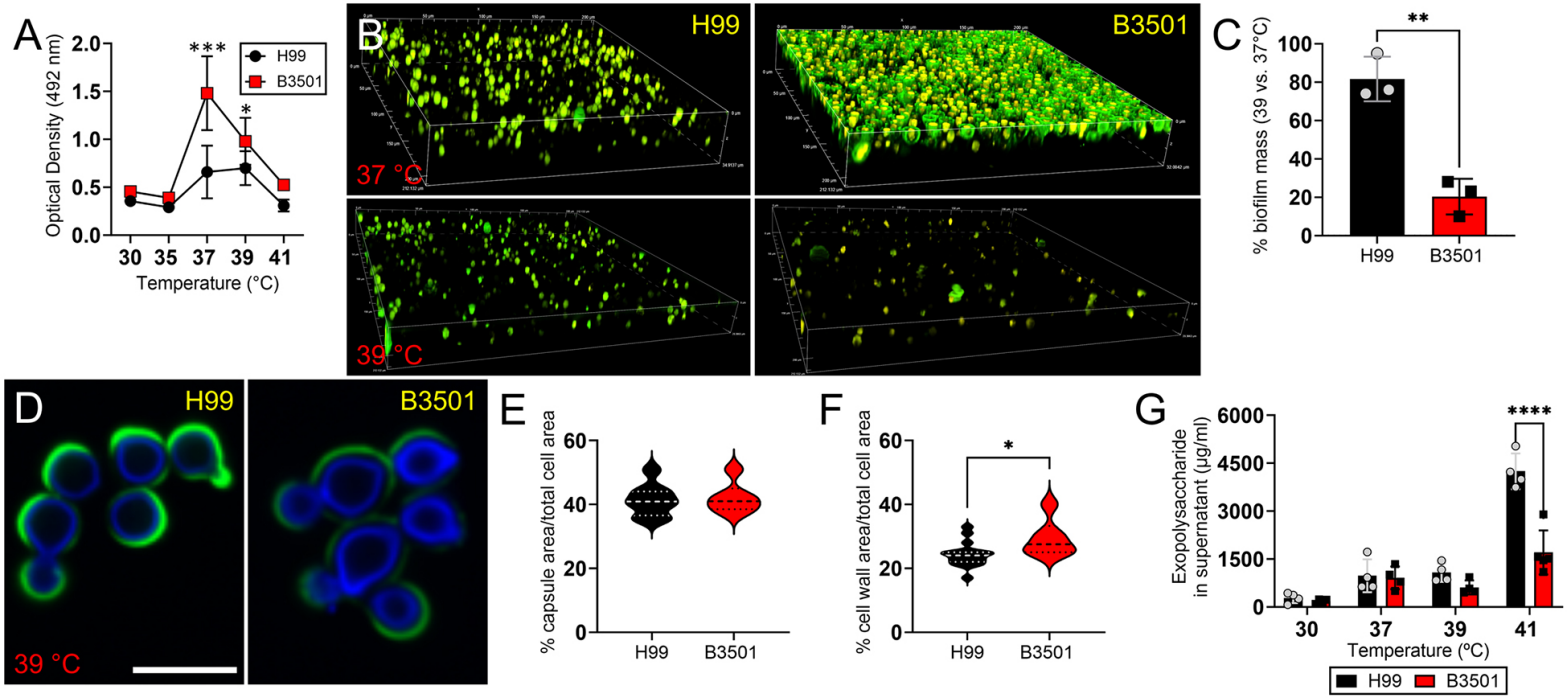
***Cd* shows considerably reduced biofilm mass compared to *Cn* at temperatures >37°C.** (A) Kinetics of cryptococcal biofilm formation in 96-well polystyrene microtiter plates at various temperatures (30, 35, 37, 39 and 41°C), as determined by XTT reduction assay. Each point represents an average of four independent measurements. (B) Confocal images of *Cn* H99 (left column) and *Cd* B3501 (right column) biofilms grown at 37°C (top row) and 39°C (bottom row) for 48 h. Mature cryptococcal biofilms were stained with FUN-1 (yellow) and GXM-specific mAb 18B7 conjugated to FITC (green). The pictures were taken using a 63× power field. (C) The percentage biofilm mass (37°C versus 39°C) was compared between *Cn* H99 and *Cd* B3501 strains. The average of three biofilm images (*n*=3; each symbol, gray circles or black squares) per strain per experiment (*n*=3) at the temperatures are shown. (D) Fluorescent images of cryptococcal cells (left, H99; right, B3501) derived from mature biofilms (48 h) grown at 39°C were taken with a confocal microscope. mAb 18B7 and Calcofluor White were used to stain the capsule (green) and cell wall (blue), respectively. The pictures were taken with a 100× power field. Scale bar: 10 μm. (E,F) The percentage capsule (E) and cell wall (F) area relative to the total cell area was determined. Fifteen cells per strain were measured. (G) The exopolysaccharide concentration in the supernatant of cryptococcal biofilms was determined using the Dubois method. Each point represents an average of four independent measurements. For A, C and E-G, bars represent the average of independent measurements. Error bars denote s.d. Significance (****P<0.0001; ***P<0.001; **P<0.01; *P<0.05) was calculated using single or multiple Student's *t*-test analyses. These experiments were performed twice, and similar results were obtained.

The biofilm-forming properties of *Cn* H99 and *Cd* B3501 strains were evaluated after growth on polystyrene microtiter plates at various temperatures (30, 35, 37, 39 and 41°C), as determined by XTT reduction assay (Fig. 6A). *Cd* B3501 formed denser biofilms than *Cn* H99 biofilms at 37°C (*P*<0.001) and 39°C (*P*<0.05) at 48 h (Fig. 6A), as indirectly measured by the metabolic activity of the adhered cells. *Cn* H99 and *Cd* B3501 biofilm-derived and planktonic cells showed no differences in viability after exposure to 39°C for 48 h (Fig. S4). Confocal microscopic images of *Cn* H99 and *Cd* B3501 biofilm growth in polystyrene 96-well plates were analyzed to determine biofilm structural development (Fig. 6B). The green fluorescence resulted from mAb 18B7 binding to yeast GXM, and the yellow fluorescence, due to FUN-1 staining, localized to dense aggregates in the cytoplasm of metabolically active cells. Consistent with the XTT assay results, *Cd* B3501 biofilms were significantly more robust than *Cn* H99 biofilms at 37°C. This was evidenced by the uniformly distributed *Cd* B3501 cells in biofilms across the plastic surface, whereas *Cn* H99 cells formed scattered clusters surrounded by empty spaces. However, the percentage biofilm mass for *Cd* B3501 strain was significantly lower at 39°C (23%) than at 37°C, whereas the *Cn* H99 strain retained 76% of its biofilm mass at this temperature (*P*<0.01; Fig. 6C). Moreover, we examined morphological changes in *Cn* H99 and *Cd* B3501 biofilm-derived cells after exposure to 39°C. For this, we stained the biofilm-derived yeast cell walls with Calcofluor White dye (blue) and the capsular GXM with mAb 18B7-conjugated to Alexa Fluor 488 (green; Fig. 6D). Next, we calculated the percentage of capsule (Fig. 6E) and cell wall (Fig. 6F) area relative to the total cell area. Similar to what was observed in planktonic cryptococci (Fig. 6D), there was no significant difference in the percentage capsule area (median: ∼40% for both) between the strains (Fig. 6E). Nevertheless, *Cd* B3501 cells (median: 29.3%) exhibited a larger percentage cell wall area compared to *Cn* H99 cells (median: 24.3%; Fig. 6F), indicating potential thermal stress for *Cd* cells. Given the impact of high temperature on *Cd* B3501 biofilm structure and biofilm-derived cell wall, we compared and quantified exopolysaccharide secretion by *Cn* H99 and *Cd* B3501 biofilms (*n*=4 per group) at increasing temperatures (30, 37, 39 and 41°C) after 48 h (Fig. 6G). H99 cryptococci exhibited significantly higher exopolysaccharide secretion than B3501 cells only at 41°C (*P*<0.0001). Our results suggest that *Cn* H99 biofilms maintain more biomass than *Cd* B3501 biofilms after exposure to 39°C or febrile temperatures, potentially providing a crucial advantage in systemic host colonization.

### *Cn* strains show higher Hsp gene expression after thermal stress than *Cd* strains

**Fig. 7.**
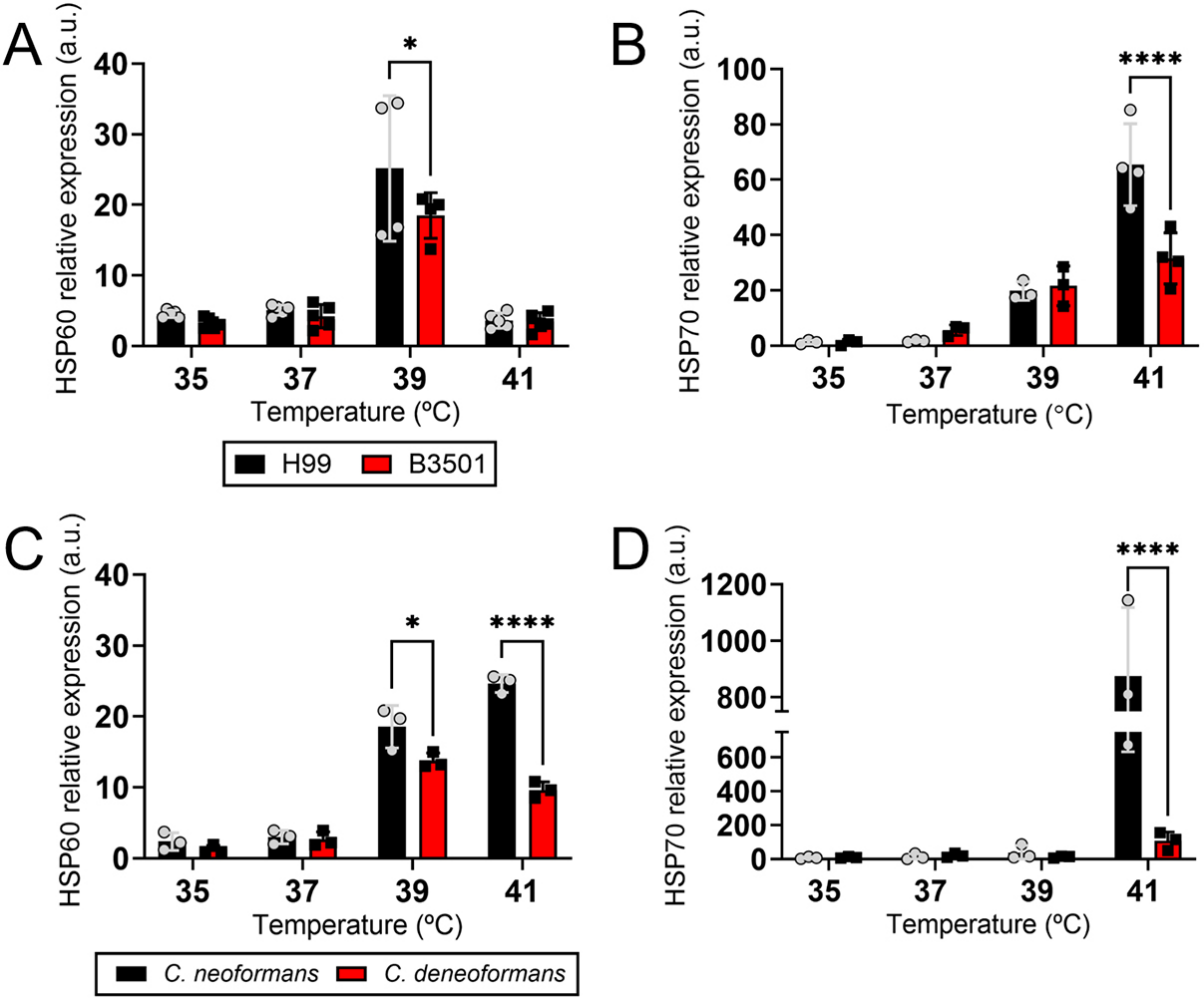
***Cn* strains have, on average, higher *hsp60* and *hsp70* expression than *Cd* strains.** (A-D) The expression of *hsp60* and *hsp70* (a.u., arbitrary units) in yeast cells from *Cn* H99 (*n*=4-5 replicates) (A), *Cd* B3501 (*n*=3-4 replicates) (B), *Cn* clinical isolate (*n*=3; 52, 55 and 62) (C) and *Cd* clinical isolate (*n*=3; 8, 9 and 11) (D) strains after exposure to increasing temperatures (35, 37, 39 and 41°C) for 1 h was determined by quantitative PCR analysis. *Gapdh* gene was used as a reference. For A-D, bars represent the average of three to five replicates/isolates, and error bars denote s.d. Significance (****P<0.0001; *P<0.05) was determined using multiple Student's *t*-test analysis. These experiments were performed twice, and similar results were obtained.

HSPs are highly conserved molecules, which are both constitutively expressed and upregulated in response to various stress conditions, particularly, high temperatures (Feder and Hofmann, 1999). Therefore, we performed relative quantification of mRNA to evaluate differences in *hsp60* and *hsp70* expression among the *Cn* and *Cd* strains after exposure to 35, 37, 39 and 41°C (Fig. 7). Given the strain-to-strain variations, particularly between standard laboratory strains (H99 and B3501) and clinical isolates, we analyzed the relative expression of each Hsp gene for standard strains separately from those of the clinical isolates. The expression of *hsp60* and *hsp70* was significantly higher in *Cn* H99 cells than in *Cd* B3501cells at 39°C (*P*<0.05; Fig. 7A) and 41°C (*P*<0.0001; Fig. 7B). Similarly, *Cn* clinical isolates (55, 59 and 62) exhibited significantly higher *hsp60* expression than that shown by *Cd* clinical isolates (8, 9 and 11) at 39°C (*P*<0.05) and 41°C (*P*<0.0001; Fig. 7C). In addition, *hsp70* was significantly more expressed in *Cn* clinical isolates than in *Cd* isolates at 41°C (*P*<0.0001; Fig. 7D). Our findings show a correlation between *hsp60* and *hsp70* upregulation and *Cn* H99 survival at elevated temperatures (≥39°C), which could help explain the systemic infection potential of *Cn* strains and the preference of *Cd* strains for cooler tissues such as the sinuses or skin.

## DISCUSSION

Skin lesions resulting from *Cn* infection are found in ∼5% of patients with cryptococcal meningoencephalitis (Kwon-Chung et al., 1992), with a higher frequency observed in liver transplant recipients receiving tacrolimus (Singh et al., 1997) or in patients infected with *Cd* strains (Dromer et al., 1996). Most often, skin lesions are attributable to hematogenous dissemination. The issue of lesions associated with a skin portal of entry (i.e. primary cutaneous cryptococcosis) has been controversial (Kwon-Chung et al., 1992). However, in addition to well-documented laboratory injuries caused by *Cn*-contaminated needles (Casadevall et al., 1994), a French nationwide study presented strong evidence suggesting that the skin should be recognized as an additional portal of entry for *Cryptococcus* (Neuville et al., 2003), particularly for *Cd* strains. To support this possibility, and given the critical role of fungal adhesion in infection and colonization, we demonstrated that *Cd* B3501 cells adhere better to human keratinocytes and melanocytes than *Cn* H99 cells. The differences in adhesive properties to human skin cells between these strains might be attributed to variations in the polysaccharide composition of the capsule. We have previously demonstrated that capsular polysaccharide isolated from *Cd* B3501 cells attaches better to polystyrene than capsular material from *Cn* H99 cells and enhances biofilm formation (Martinez and Casadevall, 2005). Similarly, the local release of capsular polysaccharide by *Cd* B3501 promotes cell adhesion and biofilm formation (Martinez and Casadevall, 2005). However, using *cap67*, a strain defective in capsular production derived from *Cd* B3501, we demonstrated a substantial reduction in human skin cell adhesion by cryptococci. Previous results using the acapsular mutant *cap59* implicated the polysaccharide capsule as an important structural component for biofilm formation (Martinez and Casadevall, 2005), which is driven by early adhesion to a biological or non-biological surface.

**Fig. 8.**
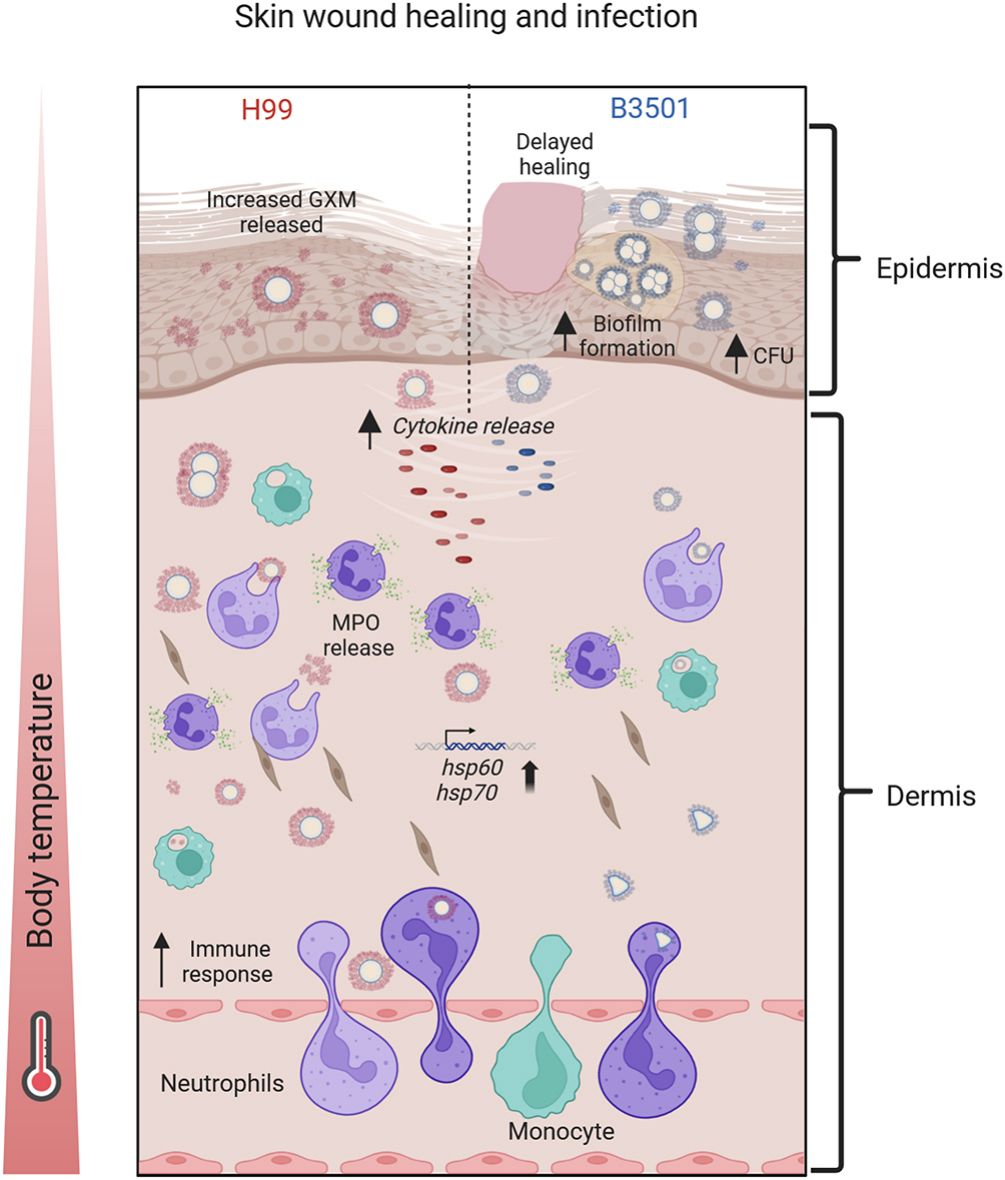
***Cn* and *Cd* skin infection pathogenesis and host responses.** In cutaneous wounds infected with *Cn* H99 strain, there is a massive increase in cellular recruitment to the damaged tissue and substantial release of immune molecules (e.g. MPO and proinflammatory cytokines) compared to that in wounds infected with *Cd* B3501 strain. High GXM release occurs in the superficial area of the wound infected by H99, whereas *hsp60* and *hsp70* are highly expressed in dermal tissue as temperature increases due to inflammation, which results in increased wound healing. In cutaneous wounds infected with *Cd* B3501 strain, robust biofilm formation on the surface of the wound takes place, facilitating fungal survival. There is also a reduction in cellular infiltration and immune molecule production, resulting in delayed wound healing. *hsp60* and *hsp70* expression in *Cd* strains is less than that in *Cn* strains. Created in BioRender by Charles-Niño, C. L. (2025). https://BioRender.com/h63r638. This figure was sublicensed under CC-BY 4.0 terms.

The association between *Cd* and cutaneous infections is linked to differences in dermatotropism and temperature tolerance between *Cn* and *Cd* strains (Martinez et al., 2001). Our results, using a cutaneous murine model of infection, suggest that these differences in thermotolerance could explain the invasive nature of the *Cn* strains, such as H99, which are found to infect the deep dermal tissue layers. In contrast, *Cd* strains, such as B3501, are predominantly associated with the stratum corneum of the epidermis (Fig. 8). In Neuville et al. (2003), evidence of cutaneous cryptococcosis in patients included the absence of dissemination and, predominantly, a solitary skin lesion on exposed areas, often presenting as a whitlow (an abscess in the soft tissue near a fingernail or toenail) or phlegmon (a localized area of acute inflammation of the soft tissues), along with a history of skin injury.

We also demonstrated that infection with the *Cd* B3501 strain delayed wound healing. This could be explained by the lower temperature at the lesion site, which could advantageously confine fungal cells to the infection site, facilitating their growth and cell-to-cell interactions, thereby promoting biofilm-like formation. Previous animal studies have shown that body temperature can influence the course and outcome of cryptococcal infection. The survival of mice with cryptococcosis is longer when they are kept at room temperatures of 35-36°C, compared to when they are exposed to cooler temperatures (Kuhn, 1939a). Chicken embryos infected with *Cryptococcus* show an enhanced survival rate when incubated at temperatures above 39°C (Klingman et al., 1951). Rabbits and pigeons, which are notably resistant to cryptococcosis, might be protected owing to their higher core body temperatures (Kuhn, 1939b). Systemic infection in rabbits often leads to a higher infection burden in the testis, which could reflect the lower temperature in the scrotum (Bergman, 1966). In humans, there is a case report of an individual co-infected with malaria and cryptococcosis who showed clearance of yeast cells from the cerebrospinal fluid during febrile episodes caused by relapsing malaria (Ashiru and Akang, 1994). Heat-sensitive cryptococcal strains have been reported to be rhinotropic, which presumably reflects a tropism of the cooler tissues of the nose (Dixon and Polak, 1986). Our high-temperature studies using clinical specimens suggest that predominance of *Cn* strains among clinical isolates can be explained by the temperatures associated with high fever, which can suppress the growth of the less thermotolerant *Cd* strains. This hypothesis is further supported by the differences in *hsp60* and *hsp70* expression observed in clinical isolates from both species, particularly after prolonged exposure to febrile-like temperatures. HSP90 is an essential chaperone required for thermotolerance in *Cryptococcus* (Fu et al., 2022). Depletion of *hsp90* transcript in *Cn* significantly decreased growth in mice (Fu et al., 2022); therefore, it would be interesting in the future to study differences in *Cn* and *Cd* HSP90 expression at elevated temperatures and its interaction with HSP60 and HSP70.

The local immune response to cryptococcal infection might be efficient enough to prevent the development of a localized infection in most individuals. Even in immunocompromised patients, the cutaneous inflammatory response is intense (Noble and Fajardo, 1972) compared with that observed in the brain (Chretien et al., 2002; Lee et al., 1996) or lungs (Curtis et al., 1994). In our wound model, the findings suggest that the *Cn* H99 strain induces a much stronger inflammatory response compared to the *Cd* B3501 strain (Fig. 8). It is conceivable that *Cn* and *Cd* have evolved distinct predilection for different infection routes, allowing these strains to adapt to various environments and to immune responses specific to certain tissues. Localized aggregation or biofilm-like formation, along with GXM release, could promote latency and prolong the survival of *Cd* strains during cutaneous infection. In our study, *Cd* biofilm formation appears to be temperature regulated, showing a robust architecture at 37°C but being significantly reduced at 39°C. Likewise, *Cd* B3501 biofilm-derived cryptococci grown at 39°C displayed a thicker cell wall relative to that of *Cn* H99 biofilm-derived cells, which could be indicative of stress related to both high-temperature exposure and biofilm reduction. In contrast, the inability of individual cryptococci to form biofilms and induce a severe inflammatory response during wound infections might exert strong selective pressure on *Cn* strains to infect the host through the respiratory tract. This could also facilitate dissemination, which could be more challenging when the cells are growing within a dense polysaccharide matrix. This hypothesis is supported by our *in vitro Cn* H99 biofilms, which were characterized by separated cryptococci uniformly distributed across the plastic substrate. *Cn* biofilms showed more structural stability than *Cd* biofilms at 39°C, likely due to higher Hsp gene expression and increased exopolysaccharide production, especially at febrile temperatures. Furthermore, *Cn* strains that invade the CNS tend to form cryptococcomas or biofilm-like structures in brain tissue, surrounded by extensive capsular polysaccharide. This phenotype could also explain the difficulty in eradicating the infection once the CNS is colonized.

The polysaccharide capsule is *Cryptococcus*’ main virulence factor and a defensive structure against environmental stress and phagocytosis. Interestingly, we found that *Cn* H99 and *Cd* B3501 strains induced capsular production differently in response to temperature. For example, *Cn* H99 cells enlarged their capsule after exposure to ≥39°C, which could provide this species with a survival advantage during systemic infection. Silveira et al. (2013) demonstrated that HSP70 is found on the surface of *Cn* H99 cells, associated with the capsule, and its presence enhances fungal binding to host cells (Silveira et al., 2013). HSP70 also protects phagocytosed *Cn* H99 cells from being killed by macrophages (Silveira et al., 2013). In contrast, *Cd* B3501 cells considerably increased their capsule size rapidly after exposure to ≥37°C, indicating that cryptococci in this species are thermosensitive and undergo stress at mammalian body temperature. Deletion of *ssa1* reduced *Cd* laccase production and melanization (Zhang et al., 2006), suggesting that HSP70 is essential for fungal virulence and stress management. For example, SSA1 deficiency impairs innate immune control of cryptococcal infection in the mouse lungs by skewing the host macrophage response toward a non-protective M2 phenotype (Eastman et al., 2015). Moreover, little is known about the function of *Cn* HSP60 in pathogenesis. Considering its significance for other fungi such as *Histoplasma capsulatum*, this chaperonin necessitates further studies to understand its importance during cryptococcal infection. HSP60 in *H. capsulatum* is a major surface adhesin to mammalian macrophages (Long et al., 2003), and studies of antibody-mediated protection against this fungus have provided insight into the complexity involving this protein (Nosanchuk et al., 2003). The importance of HSP60 in *H. capsulatum* biofilm formation and infection of wax-moth *Galleria mellonella* larvae (Fregonezi et al., 2020) highlights the possible involvement of this protein in cryptococcal adhesion and biofilm formation *in vivo*.

We considered sex as a biological variable in our studies by investigating the wound-healing and anti-cryptococcal responses in mice infected with the cryptococcal strains. The overall temperature of post-pubertal female mice is 0.2-0.5°C higher than that of male mice, particularly when progesterone levels, and to a lesser extent, estradiol levels, are elevated during the estrous cycle (Sanchez-Alavez et al., 2011). This is especially important because human immunodeficiency virus (HIV)-infected males with cryptococcal meningoencephalitis are more susceptible to developing cerebral infarcts, suffer mental health alterations and die of cerebral cryptococcosis (Mishra et al., 2018). Similarly, immunocompromised females with anemia are more susceptible to fatal cryptococcal meningoencephalitis in Africa (Stadelman et al., 2021). Female mice exhibited faster wound healing both at baseline and in the presence of cryptococcal infection, regardless of the species. Many studies have shown that cutaneous wounds heal more slowly in males than in females, and that the healing response differs between the sexes (Ashcroft, 2004). The impact of hormones on cellular and tissue responses has major downstream effects on the rate of healing and cryptococcal infection clearance (Guess et al., 2018), resulting from enhanced inflammation, impaired cytokine signal transduction, and an altered balance of protein production and degradation. For instance, *Cn* clinical isolates from HIV-infected females showed longer doubling times and more GXM released *in vitro* than those from HIV-infected males (McClelland et al., 2013). However, GXM release was associated with the presence of testosterone but not estradiol. Although we did not compare the differences in GXM release in wounds from male and female mice, this polysaccharide being immunosuppressive and potentially stimulated by testosterone might impair cutaneous responses to healing and infection in male mice. Incidentally, even though human female-derived macrophages phagocytosed more cryptococci than male-derived macrophages *in vitro*, male-derived macrophages showed a higher cryptococcal intracellular load but increased cryptococcal killing (McClelland et al., 2013). HIV-infected males with cryptococcosis in endemic regions exhibited higher median pro- and anti-inflammatory, monocyte/macrophage differentiation, maturation, migration, immune exhaustion and cytotoxicity cytokines than HIV-infected females with cryptococcosis in the same regions (Stadelman et al., 2021). Further studies are necessary to establish sex-associated differences during cutaneous, pulmonary and systemic cryptococcal infections.

In summary, the *Cd* B3501 strain is more dermatotropic than the *Cn* H99 strain, likely due to differences in their pathobiology and thermotolerance. The results of the present study demonstrate that *Cn* strains are more thermotolerant, and their association with increased *hsp60* and *hsp70* expression after exposure to mammalian and high temperature support the increasing body of data that indicate major differences existing between *Cn* and *Cd* isolates in routes of infection and interactions with host surveillance cells including inflammatory responses*.* Our findings further validate the recent classification of these strains as separate species, *Cn* (serotype A) and *Cd* (serotype D) (Gago et al., 2017; Hagen et al., 2015). Moreover, given the changing climate, continued assessment of *Cryptococcus* species distribution is crucial, as thermotolerant species are expected to outcompete others.

## MATERIALS AND METHODS

### Fungi

*Cn* strain H99 was obtained from Arturo Casadevall (Johns Hopkins Bloomberg School of Public Health, Baltimore, MD, USA) and isolated by John Perfect (Duke University School of Medicine, Durham, NC, USA). *Cd* strain B3501 was acquired from the American Type Culture Collection (ATCC). The *cap67* gene deletion mutant strain of *Cd* B3501 that fails to produce capsular GXM but synthesizes small traces of other capsular components was generated by K. J. Kwon-Chung (National Institutes of Health, Bethesda, MD, USA). In addition, a total of 12 clinical isolates (*Cn* strains: 55, 59, 62, 83, 129, sm; *Cd* strains: 8, 9, 11, 13, 14, 16; a gift from Arturo Casadevall) were included in this study. MLST was performed using the International Society of Human and Animal Mycology consensus MLST scheme for *Cryptococcus*, which includes seven genetic loci – *cap59*, *gpd1*, *lac1*, *plb1*, s*od1* and *ura5* genes, and the IGS1 region (Meyer et al., 2009). The fungi were inoculated in Sabouraud dextrose broth (Difco) and incubated at 30°C for 24 h in an orbital shaker incubator set at 150 rpm.

### Ethics statement

All animal studies were approved and conducted according to the experimental practices and standards approved by the Institutional Animal Care and Use Committee at the University of Florida (Protocol: 202011067).

### Wound infection model

To investigate the propensity of *Cryptococcus* species strains to infect cutaneous tissues, male and female Balb/c mice (6-8 weeks; Charles River Laboratories) were anesthetized [ketamine (100 mg/kg)-xylazine (20 mg/kg) cocktail], their back hair was removed, and the exposed skin was cleansed with an application of iodine. Single full-thickness 5 mm-diameter wounds were created using a punch biopsy (Tru-Punch, Sklar Instruments). *Cn* H99 or *Cd* B3501 strains were then immediately inoculated onto the wounded tissue using 10^7^ cells/ml in a 25 μl suspension of the yeast prepared in sterile saline. Uninfected, wounded mice were used as controls. The wounds were photographed daily with a digital camera. Calipers were also used to measure the diameter of the wound daily, and this function was performed by two independent investigators unaware of the infecting strain. The average body temperature for all the groups of mice during this experiment was 37°C. Mice at 3 dpi were euthanized, and tissue biopsies were collected.

### CFU determinations

Tissue from the affected wound was excised at 3 dpi and homogenized in sterile phosphate-buffered saline (PBS). Serial dilutions were plated on Sabouraud dextrose agar (Difco), incubated at 30°C for 48 h, and measured for fungal colony counts normalized to tissue weights.

### Capsule measurement

The percentage capsule size (in volume) relative to the total size of the cell was determined for each cryptococcal strain obtained from tissue homogenates (Rivera et al., 1998). Ten-microliter aliquots were spotted on microscope slides, mixed with India ink (Gibco) and examined using a Leica DMi8 inverted microscope at a magnification of 40×, prior to imaging with a Leica DFC7000T digital camera using the Leica software platform LAS X. The diameter (*D*) of the whole cell (*D*_wc_) and the cell body (*D*_cb_) were each measured, and the capsule width was defined as the difference between *D*_wc_ and *D*_cb_ diameters (Chrisman et al., 2011). The volume of the whole cell (*V*_wc_) and the cell body (*V*_cb_) were calculated using the equation for the volume of a sphere, 4/3 π (*D*/2)^3^, and % capsule size relative to the total cell size was calculated using *V*_c_/*V*_wc_×100, where ‘*V*_c_’ is the volume of the capsule.

### Histological examinations

Infected tissues were excised from euthanized mice at 3 dpi, fixed in 10% formalin for 24 h, processed and embedded in paraffin. Four-micrometer sections were fixed to glass slides, and stained with H&E, MC and MPO to examine tissue morphology, fungal morphology and neutrophil infiltration, respectively. Fungal capsular polysaccharide was stained using the GXM-binding mAb 18B7 (a gift from Arturo Casadevall). Slides were first blocked with PBS supplemented with 1% bovine serum albumin (BSA) followed by the addition of IgG_1_ GXM-binding mAb 18B7 (2 μg/ml) for 1 h at 37°C. Peroxidase-conjugated goat anti-mouse IgG_1_ (Southern Biotech; 1:250) was added for 1 h at room temperature (RT) before developing with 3,3-diaminobenzidine tetrahydrochloride and 0.3% H_2_O_2_ for 5 min at RT. Slides were examined by light microscopy.

### *Cn* capsular polysaccharide determinations

*Cn* capsular GXM released in 1 ml homogenate of infected skin tissue was quantitatively measured by capture ELISA as described (Martinez et al., 2004).

### Cytokine and MPO determinations

Wound lesions were excised at 3 dpi and homogenized in PBS with protease inhibitors (Complete Mini, Roche). Cell debris was removed by centrifugation at 6000 ***g*** for 10 min. Samples were stored at −80°C until testing.

#### Cytokines

Supernatants were tested for TNFα, IFN-γ, IL-1β and IL-6 by ELISA (BD Biosciences) with a detection limit of 15.6 pg/ml.

#### MPO

Supernatants were tested for MPO by ELISA (Hycult Biotechnology) with a detection limit of 1 ng/ml.

### Fungal adhesion to human skin cells

#### CCD-1106 KERTr dermal keratinocytes

CCD-1106 KERTr (CRL-2309, ATCC) is a keratinocyte cell line isolated from skin. For the adhesion experiment, 10^5^ keratinocytes were cultured in Keratinocyte-Serum Free Medium (Gibco) [supplemented with bovine pituitary extract (Gibco), 35 ng/ml human recombinant epidermal growth factor (Gibco), 100 U/ml penicillin (Gibco) and 100 µg/ml streptomycin (Gibco)] with 10^6^ H99 or B3501 cells for 2 h at 37°C in 5% CO_2_ and washed 3× with PBS. Fluorescent microscopy was performed to visualize fungal adhesion.

#### SK-MEL-28 skin melanoma cells

SK-MEL-28 (HTB-72, ATCC) is a human skin melanoma-like cell lineage. For adhesion experiments, 10^5^ melanoma-like cells were cultured in Dulbecco's modified Eagle's medium (DMEM; Mediatech) [supplemented with 20% NCTC-109 (Life Sciences), 10% fetal bovine serum (FBS; Bio-Techne), 1% non-essential amino acids (Mediatech), penicillin-streptomycin (100 IU/ml; Life Sciences)] with 10^6^ H99, B3501 or *cap67* cells for 2 h at 37°C in 5% CO_2_ and washed 3× with PBS. Epithelial cells were washed to remove unattached fungal cells and lysed with cold water. Resulting *Cn* and *Cd* suspensions were plated onto Sabouraud dextrose agar for CFU determinations after 48 h. *Cn* and *Cd* clinical isolates were also separately tested.

### Fluorescent microscopy

CCD 1106 KERTr monolayers were fixed on circular glass coverslips (18 mm; Thermo Fisher Scientific) with 3% paraformaldehyde (Thermo Fisher Scientific) in PBS for 10 min at RT. The cells were washed with PBS-GSA (1× PBS containing 10 mM glycine and 0.2% sodium azide; Thermo Fisher Scientific) and permeabilized for 3 min with PBS-GSA containing 0.5% Triton X-100 (Sigma-Aldrich), washed again and blocked with 1% BSA in PBS-GSA for 5 min at RT. After blocking, the cells were washed 3× with 5% Tween 20 in PBS and incubated with anti-β tubulin (melanoma cell body; rabbit polyclonal; 1:50 dilution; Proteintech) conjugated to Alexa Fluor 488 (green; 1:200 dilution; Invitrogen) and anti-GXM (1:200 dilution; 18B7) conjugated to Alexa Fluor 555 (red; 1:200 dilution; Invitrogen), respectively, in an orbital shaker at 150 rpm and 37°C for 1 h. The samples were washed 3× with blocking buffer and incubated with 4′,6-diamidino-2-phenylindole (DAPI; blue; Thermo Fisher Scientific) for 1 h at 37°C. The slides were washed 3× with PBS, coverslips were affixed, and each sample was viewed to determine the adhesion of cryptococci to the cell surface (*n*≥50) with a Zeiss LSM 700 Confocal Laser Scanning Microscope. Images were collected at 40× magnification using an AxioCam digital camera and analyzed using Zen Lite digital imaging software.

### Thermotolerance of cryptococcal strains

#### Real-time growth curve

The growth of *Cn* H99 and *Cd* B3501 cells in Sabouraud dextrose broth at 30, 37, 39 and 41°C for 72 h was assessed in real time (an optical density of 600 nm measurement was taken every 2 h) for 72 h using a Bioscreen C system (Growth Curves USA).

#### CFU survival

A 0.1 ml aliquot of a 10^7^ yeast suspension (*Cn* and *Cd* clinical isolates; Table S2) was placed in a microcentrifuge tube and inserted into a pre-warmed digital dry water bath at 30, 35, 37, 39 or 41°C for 30 min. The cell suspension was then plated on Sabouraud dextrose agar and incubated for 48 h at 30°C for CFU quantification. The percentage survival was determined by comparing colonies from heat-exposed and unexposed suspensions.

#### Growth on agar

The thermotolerance of *Cn* (H99 and 83) and *Cd* (B3501 and 8) strains was evaluated on Sabouraud dextrose agar. Plates were inoculated with 10 μl drops containing 10^6^ cells in PBS, and each strain was grown in parallel to the others at 39°C for 24, 48 and 72 h to confirm colony growth.

### Fungal biofilm formation

Cryptococcal biofilms were grown in polystyrene 96-well plates (Corning) at 37°C and 39°C for 48 h (Martinez and Casadevall, 2005). The XTT reduction assay and CFU determinations (Martinez and Casadevall, 2007) were performed to measure biofilm metabolic activity and viability, respectively.

### Planktonic cell growth

Cryptococci were grown in minimal medium for 48 h at 39°C in an orbital shaker incubator set at 150 rpm, collected by centrifugation (1600 ***g***, 5 min), washed twice with PBS and counted with a hemacytometer before each experiment. As previously described and determined using the XTT reduction assay (Martinez and Casadevall, 2007), we used a density of 5×10^6^ planktonic cells for comparison to the biofilms.

### Confocal microscopy

Fungal biofilms grown at 37°C and 39°C in glass-bottom plates were incubated for 45 min in 75 μl Tris-buffered saline (TBS; Thermo Fisher Scientific) containing FUN-1 (10 μM; Molecular Probes). Wells were blocked with TBS (1% BSA) and incubated with GXM-specific mAb 18B7 (2 μg/ml). Fluorescein isothiocyanate (FITC; Molecular Probes)-conjugated goat anti-mouse (GAM)-IgG_1_ at 1 μg/ml in TBS (1% BSA) was applied. Between steps, the wells were washed with 0.05% Tween 20 (Thermo Fisher Scientific) in TBS. All incubations were done at 37°C for 1 h. FUN-1 (excitation, 470 nm; emission, 590 nm) is converted to orange-red intravacuolar structures by metabolically active cells, whereas mAb 18B7, when bound by FITC-conjugated GAM IgG_1_ (excitation, 488 nm; emission, 530 nm), labels GXM and fluoresces green. Depth measurements across the width of the device were taken at regular intervals using an upright Leica TCS SP5 confocal laser scanning microscope, and a series of horizontal (*X*-*Y*) optical sections with a thickness of 1.175 μm were taken throughout the length of the biofilm. The percentage biofilm mass was determined by comparing biofilm thickness after growth at 39°C versus 37°C using the following equation: % biofilm mass=biofilm thickness at 39°C/biofilm thickness at 37°C×100. The averages of three biofilm images (*n*=3) per strain per experiment (*n*=3) at the temperatures are shown.

### Percentage capsule and cell wall area relative to total cell area determinations

*Cn* and *Cd* biofilm-derived yeast cells were obtained after 48 h incubation at 39°C. A solution of 3.5% paraformaldehyde in PBS (pH 7.4) was used to fix the fungal cells for 20 min at RT. Calcofluor White (100 μg/ml; Molecular Probes) was added to the yeast cells to stain their cell wall following incubation at 30°C for 40 min. Next, samples were blocked with PBS+0.05% Tween 20 (1% BSA) for 30 min. Then, the fungal capsule was immunostained with GXM-specific mAb 18B7 (IgG_1_; 1 μg/ml; a generous gift from Arturo Casadevall) and FITC (Molecular Probes)-conjugated GAM-IgG_1_ (1 μg/ml) in PBS (1% BSA) for 30 min at RT. Microscopic examinations and measurements (μm) of cryptococcal biofilm-derived cell capsule and cell wall seeded on glass-bottom plates were performed by precisely tracing each structure using a Nikon Eclipse Ti2 inverted confocal laser scanning microscope and the NIS-Elements software. The percentage of the capsule (green) and cell wall (blue) area relative to the total cell area were calculated using the following equation: % capsule or cell wall area=capsule or cell wall area/whole cell area×100.

### Quantification of fungal biofilm exo-polysaccharide using the phenol-sulfuric acid method

Biofilm supernatant was analyzed as described by DuBois et al. (1956), with a final volume of 10 ml, using pure supernatant, 7.5 ml supernatant+2.5 ml pyrogen-free water, 5 ml supernatant+5 ml pyrogen-free water, and 2.5 ml supernatant+7.5 ml pyrogen-free water.

### RNA extraction, cDNA synthesis and quantitative PCR

We assessed fungal *hsp60* (forward, 5′-GCCGGCTGCAACCCCATGGA-3′; reverse, 5′-AGGGGAGATGAAGCCTCGGTCG-3′) and *hsp70* (forward, 5′-TGATCCAGGTCTTCGAGGGT-3′; reverse, 5′-TACGTTAGGCGAAGGTTCCT-3′) expression after 60 min of heat exposure. RNA extraction from a 5 ml (1×10^7^ cells/ml) yeast culture suspension was performed using a Quick-RNA fungal/bacterial extraction kit (Zymo Research), following the manufacturer's instructions; 200 ng of total RNA was used to synthesize cDNA with a Verso cDNA Synthesis kit (Thermo Fisher Scientific). The control reaction was set up using all components of the reaction mixture but without the reverse transcriptase enzyme. The expression of genes was determined by quantitative PCR using PowerUp SYBR Green Master Mix 2X (Applied Biosystems). A no-template control and a no-reverse transcriptase control were included. Glyceraldehyde-3-phosphate dehydrogenase (*Gapdh*) was used as the reference gene (forward, 5′-GCTGCYGCTAACATCATCCC-3′; reverse, 5′-YGAAATCAGTRGAGACAACA-3′). Relative expression was determined using the 2^−DDCT^ method on a qTOWER^3^G system (Analytik Jena). Reactions were set up using 300 nM primers and 2.5 μl of the cDNA template (diluted 1:10). Cycling conditions were as follows: 50°C for 2 min, 95°C for 2 min, and then 40 amplification cycles of 95°C for 15 s, 56°C for 30 s, and 72°C for 30 s. The samples were cooled to 55°C, and a melting curve for temperatures between 55°C and 95°C, with 0.5°C increments, was recorded. All reactions were carried out in triplicate. Target gene expression was measured using expression relative to that of the *Gapdh* reference gene and the cryptococci grown at 30°C reference sample. Data analysis was carried out using qPCRsoft (Analytik Jena).

### Statistical analysis

All data were subjected to statistical analysis using Prism 10.1.3. (GraphPad Software). *P*-values for individual or series of comparisons were calculated using paired two-tailed Student's *t*-test or multiple Student's *t*-test analyses, respectively. *P*-values for multiple comparisons were calculated by one-way analysis of variance (ANOVA) and were adjusted by Tukey's post-hoc analysis. *P*<0.05 was considered significant.

## Supplementary Material

10.1242/dmm.052141_sup1 Supplementary information
