# Supplementary material for: Reduced growth and biofilm formation at high temperatures contribute to Cryptococcus deneoformans dermatotropism
Source: Dis Model Mech. 2025 Mar 25;18(9):dmm052141. doi: 10.1242/dmm.052141 (PMC11972076; doi:10.1242/dmm.052141)
Supplement: Supplementary information [file dmm-18-052141-s1.pdf]

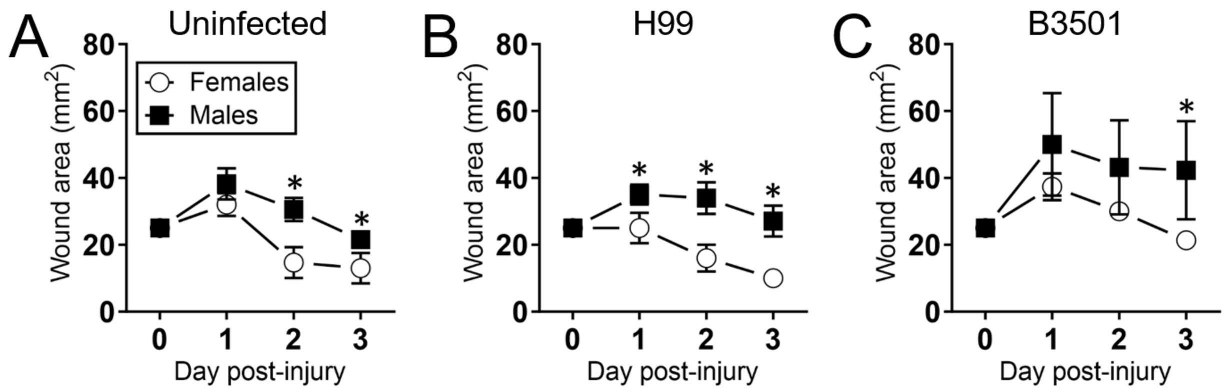

Fig. S1. Uninfected and infected female mice show faster wound healing than male mice. Wound size analysis of **(A)** uninfected and **(B)** *Cn* H99- or **(C)** *Cn* B3501-infected female and male Balb/c mice skin lesions. Time points are the averages of the results for five different wound measurements (n = 5 mice per group; a single wound per mouse), and error bars denote standard deviations (SDs). Significance (P < 0.05) was calculated by multiple student's *t*-test analysis. Asterisk (*) indicates significantly higher than female mice. The biological sex comparisons were performed with the same data presented in Fig. 1.

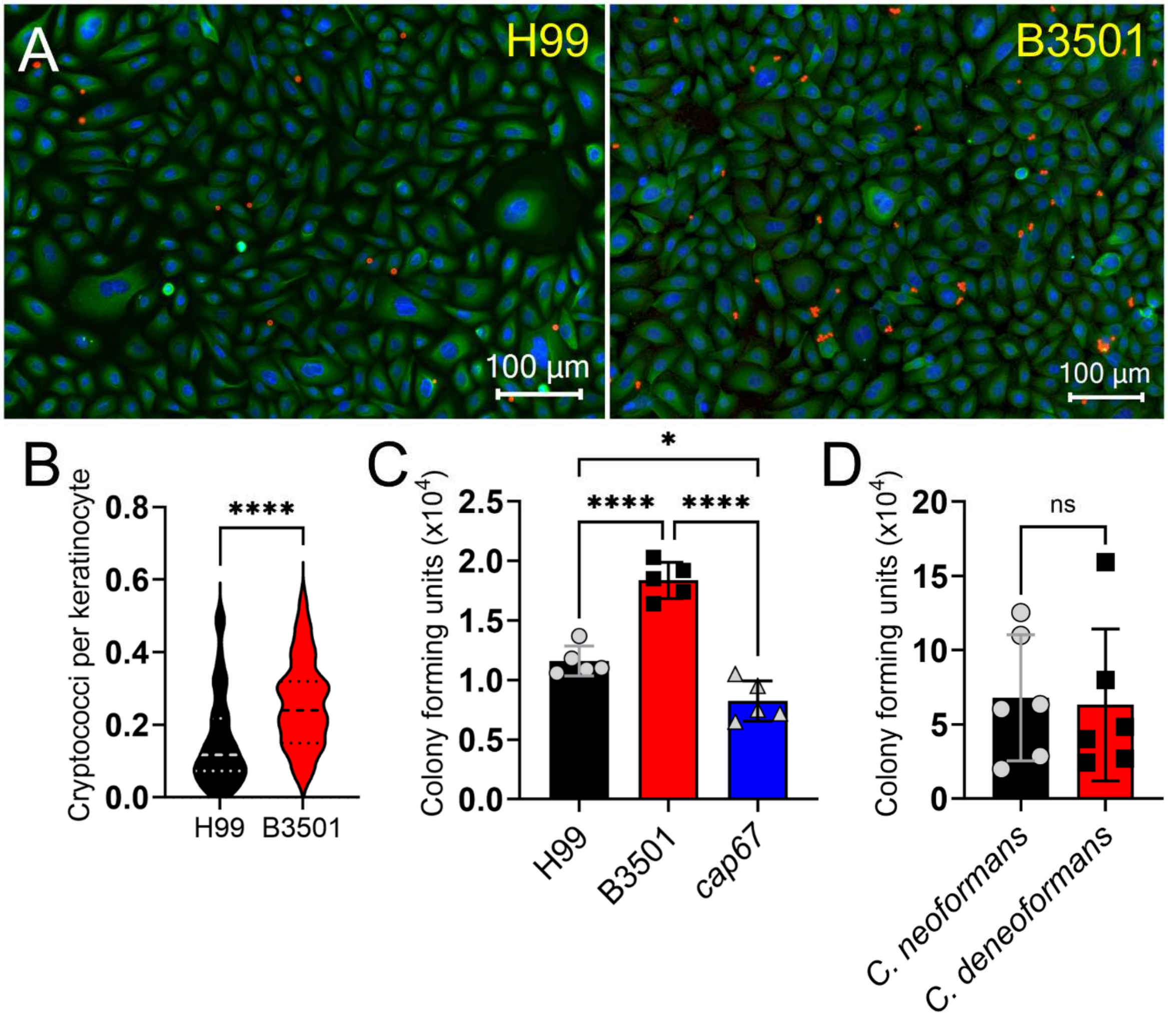

Fig. S2. *Cd* significantly adheres to human skin cells. (A) Fluorescent images of *Cn* H99 and *Cd* B3501cells interacting with human dermal keratinocytes (CCD-1106 KERTr). After 4 h co-incubation at 37 °C, keratinocytes were washed and incubated with DAPI and β-tubulin to label the nuclei (blue) and cell body (green), respectively. Cryptococci were incubated with mAb 18B7-rhodamine-conjugated goat anti-mouse IgG_1 stained to label the capsular polysaccharide (red). The pictures were taken using a 20X power field. Scale bar, 100 µm. **(B)** Adhesion of H99 and B3501 cryptococci to CCD-1106 KERTr cells was determined by counting using an inverted microscope. Counting was blindly performed by 3 independent investigators. Violin plots show the averages (dashed lines) of the number of fungi attached to individual keratinocytes ($n \geq 50$ per

Disease Models & Mechanisms • Supplementary information

group), and error bars denote SDs. Attachment of **(C)** standard or **(D)** clinical fungal cells to SK-MEL-28 skin melanoma cells was determined by CFU. The capsular mutant *cap67* strain was used as a negative control when *Cn* H99 and *Cd* B3501 strains were tested. Each symbol (grey circles, black squares, or grey triangles) represents individual CFU per well (standard, $n = 5$ per group; clinical, $n = 6$ isolates per group). Bars represent the average and error bars denote SDs. Significance (****$P < 0.0001$; ***$P < 0.001$) calculated by one-ANOVA and adjusted by use of the Tukey's post-hoc analysis. ns denotes comparisons which are not statistically significant.

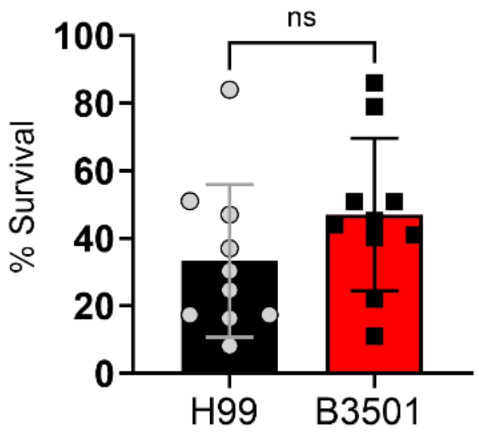

Fig. S3. *Cn* and *Cd* show no differences in viability after exposure to 41°C for 6 h. The percentage survival of *Cn* and *Cd* strains was determined by dividing the number of CFU grown on Sabouraud dextrose agar at high temperature (41°C) over those grown at optimal temperature (30°C) x 100. Each bar represents an average of 10 replicates (grey circles and black squares) per strain, and error bars denote SDs. Significance ($*P < 0.05$) was calculated using student's *t*-test analysis. ns denotes comparisons which are not statistically significant. These experiments were performed twice, and similar results were obtained.

Disease Models & Mechanisms • Supplementary information

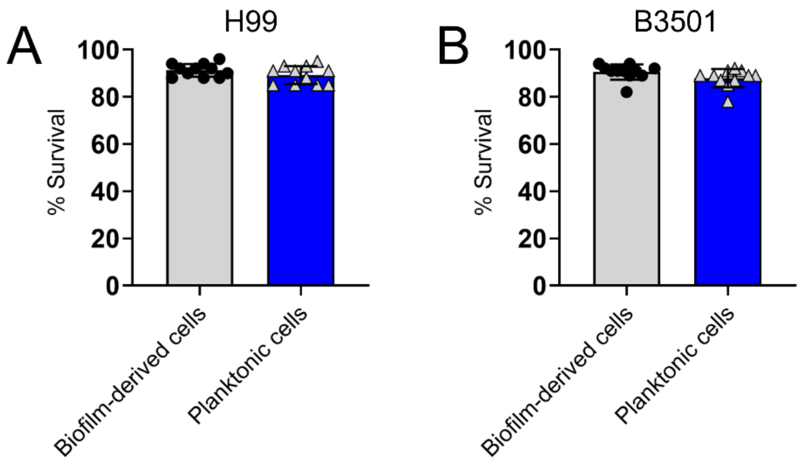

Fig. S4. *Cn* and *Cd* biofilm-derived and planktonic cells show no differences in viability after exposure to 39°C for 48 h. The percentage survival of **(A)** *Cn* H99 and **(B)** *Cd* B3501 biofilm-derived and planktonic cells was determined by dividing the number of CFU grown on Sabouraud dextrose agar at high temperature (39°C) over those grown at optimal temperature (30°C) x 100. Each bar represents an average of 10 replicates (black circles and grey triangles) per condition per strain, and error bars denote SDs. Significance (*$P < 0.05$) was calculated using student's *t*-test analysis. These experiments were performed twice, and similar results were obtained.

Disease Models & Mechanisms • Supplementary information

Table S1. Pro-inflammatory cytokine levels in wounds of Balb/c mice infected with *Cn* and *Cd* strains.

	Cytokine levels (pg/mL) Average ± STDEV			
	**TNF-α**	**IFN-γ**	**IL-1β**	**IL-6**
**Uninfected**	191.7 ± 14.6	4.9 ± 6.5	59.6 ± 8.4	489.4 ± 15.3
**H99**	355.2 ± 10.2*	65 ± 4.8**a*	867.8 ± 8.6**a*	2092.1 ± 13.2**a*
**B3501**	172.3 ± 14	27.4 ± 4.5	610.8 ± 13.9*	778.7 ± 18.4

n = 5 mice per group; a single wound per mouse. Value is significantly greater (*) or lesser (†) than the value for uninfected mice ($P < 0.05$). Value is significantly greater (*a*) than the value for B3501 infected mice ($P < 0.05$).

Table S2. Multilocus sequence typing for *Cryptococcus* species clinical isolates used in this study.

Species	Strain	*cap59*	*gpd1*	*igs1*	*lac*	*plb1*	*sod1*	*ura5*	Seq.
Cn	55	1	1	25	3	2	1	1	77
	59	1	23	25	3	4	1	1	95
	62	17	23	25	3	2	1	2	258
	83	1	23	25	4	4	1	1	257
	129	24	23	28	3	2	1	2	259
	sm	16	23	24	3	4	1	19	260
Cd	8	7	1	1	2	1	1	2	23
	9	1	1	25	3	2	1	1	77
	11	16	1	28	2	13	1	19	253
	13	9	1	14	6	4	1	16	254
	14	16	1	53	2	14	1	20	255
	16	17	1	28	6	14	1	16	256

Disease Models & Mechanisms • Supplementary information
